# Supplementary material for: HIV-Tocky system to visualize proviral expression dynamics
Source: Commun Biol. 2024 Mar 20;7:344. doi: 10.1038/s42003-024-06025-8 (PMC10954732; doi:10.1038/s42003-024-06025-8)
Supplement: Supplementary file 2 — Supplementary Information [file 42003_2024_6025_MOESM2_ESM.pdf]

Supplementary Figures for
HIV-Tocky system to visualize proviral expression dynamics

Omnia Reda, Kazuaki Monde, Kenji Sugata, Akhinur Rahman, Wajihah Sakhor, Samiul Alam Rajib, Sharmin Nahar Sithi, Benjy Jek Yang Tan, Koki Niimura, Chihiro Motozono, Kenji Maeda, Masahiro Ono, Hiroaki Takeuchi, Yorifumi Satou

Corresponding author: Yorifumi Satou
1-1-1 Honjo, Chuo-ku, Kumamoto, 860-8556, Japan
Tel: +81-96-373-6830
Email: y-satou@kumamoto-u.ac.jp

This PDF file includes:

Supplementary Figures S1 to S16

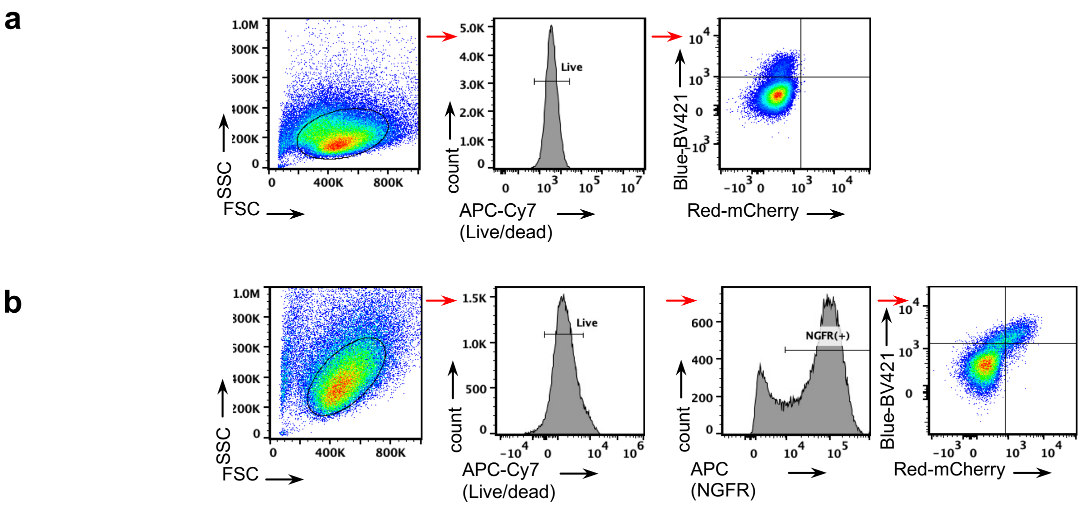

Supplementary Fig. 1. Gating strategy for Timer-FP detection by flow cytometry
a Gating strategy for Timer-FP in cells infected with HIV_{Timer}
b Gating strategy for Timer-FP in cells infected with HIV_{TNGFR}

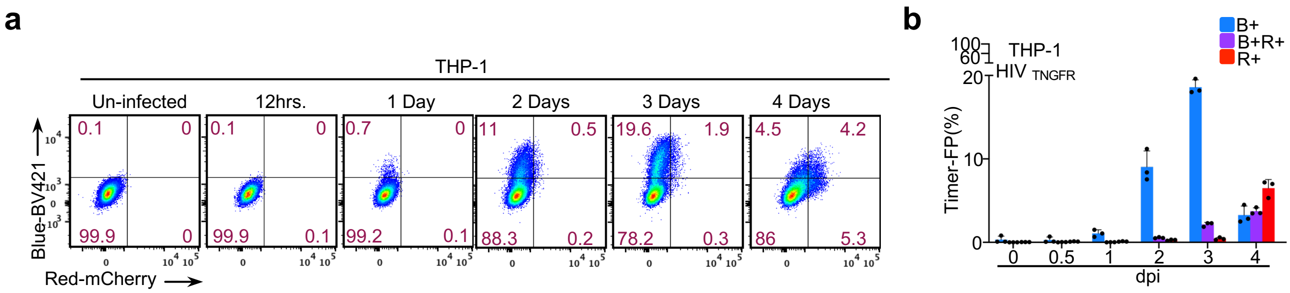

Supplementary Fig. 2. Establishment of *in vitro* HIV-Tocky reporter system in THP-1 cell line
a Representative flow plots from time course infection of HIV_{TNGFR} in THP-1 cell line. Cells were infected by HIV_{TNGFR} virus adding and followed up for Timer expression until day 4 post-infection. **b** Representative bar graphs denoting the percentage of each Timer population from THP-1 infection experiment (n = 3 biologically independent experiments, mean ± SD).

a

b

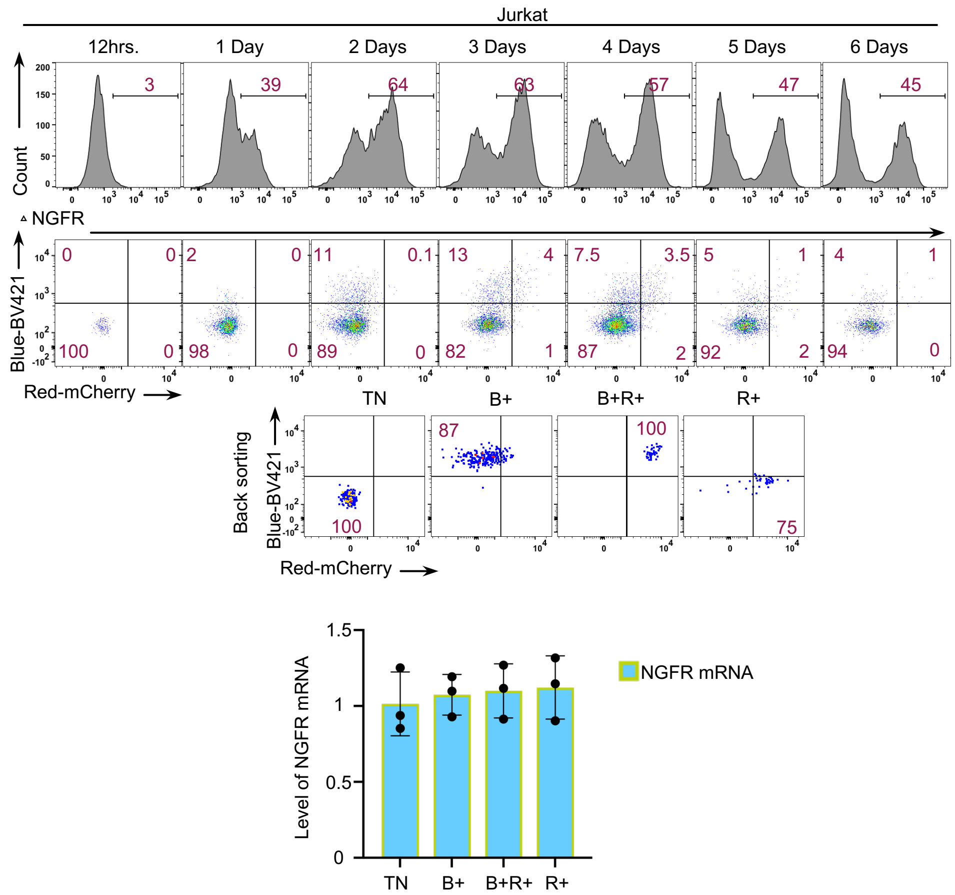

Supplementary Fig. 3. Jurkat infection and cell sorting for viral transcript quantification
a HIV_{TNGFR} infection into Jurkat T cells 12 hrs. to 6 dpi; the upper panel shows NGFR+ cells, the middle panel shows Timer populations' distribution, and the lower panel shows purity check of sorted Timer populations (TN, B+, B+R+, and R+). **b** Total RNA isolated from each Timer population of Jurkat-infected cells with HIV_{TNGFR} used in Figure **1h** was subjected to SYBR green RT-qPCR analysis. NGFR mRNAs were quantified relative to cellular GAPDH and fold change in NGFR expression was calculated relative to its level in the TN population. (n = 3 biologically independent experiments, mean ± SD).

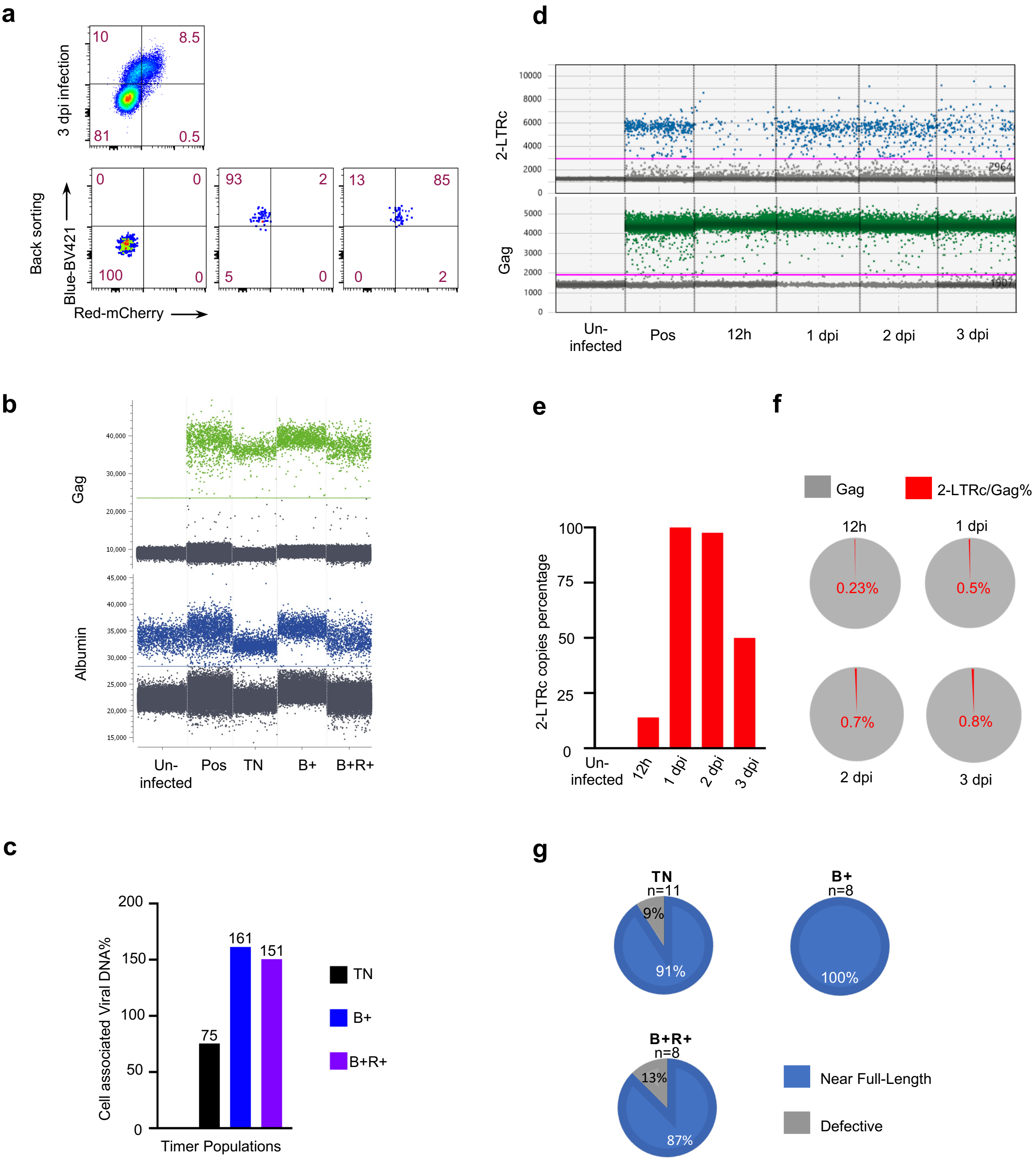

Supplementary Fig. 4. **a** HIV_{Timer} infection into Jurkat T cells at 3 days post-infection. The sample used for sorting Timer fractions (TN, B+, and B+R+). **b** Cell-associated viral DNA result by ddPCR. 1D dot-plot showing droplet fluorescence intensity on the y-axis and each sample tested on the x-axis for *Gag* gene (upper panel) and *Albumin* gene (lower panel); pos is the assay positive control. **c** Cell-associated DNA for each Timer population was calculated by the following formula (copy number of HIV-1 *gag* DNA)/[(copy number of Albumin)/2] x100. **d** 1D dot plot showing droplet fluorescence intensity on the y-axis and each sample tested on the x-axis for 2-LTRc (upper panel) and *Gag* gene (lower panel). **e** 2-LTRc dynamics by quantifying copies/uL from ddPCR data shown in **d** starting at 12 h post-infection until 3 dpi. Copies/ul per time point were divided by copies retrieved on 1 dpi and plotted as a percentage. **f** Pie charts showing the percentage of 2-LTRc to total *Gag* per time point for data shown in **e**. **g** Pie charts reflecting the proportion of defective and intact proviruses presented in **c**; n= total number of provirus sequences analyzed.

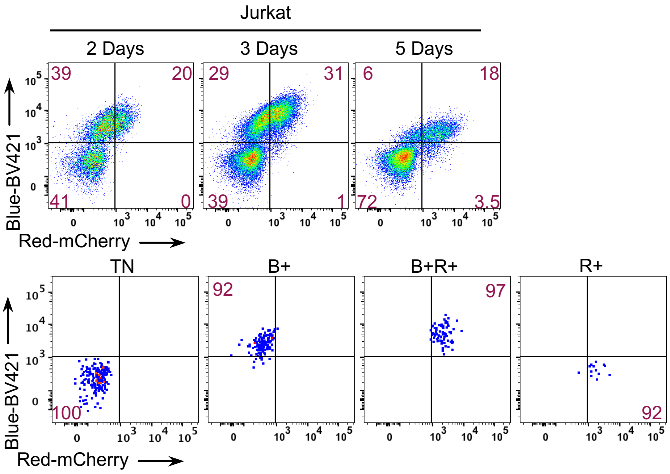

Supplementary Fig. 5. Jurkat infection for IS analysis

HIV_{Timer} infection into Jurkat T cells 2, 3, and 5 dpi; the upper panel shows Timer populations' distribution, and the lower panel shows purity check of sorted Timer populations (TN, B+, B+R+, and R+).

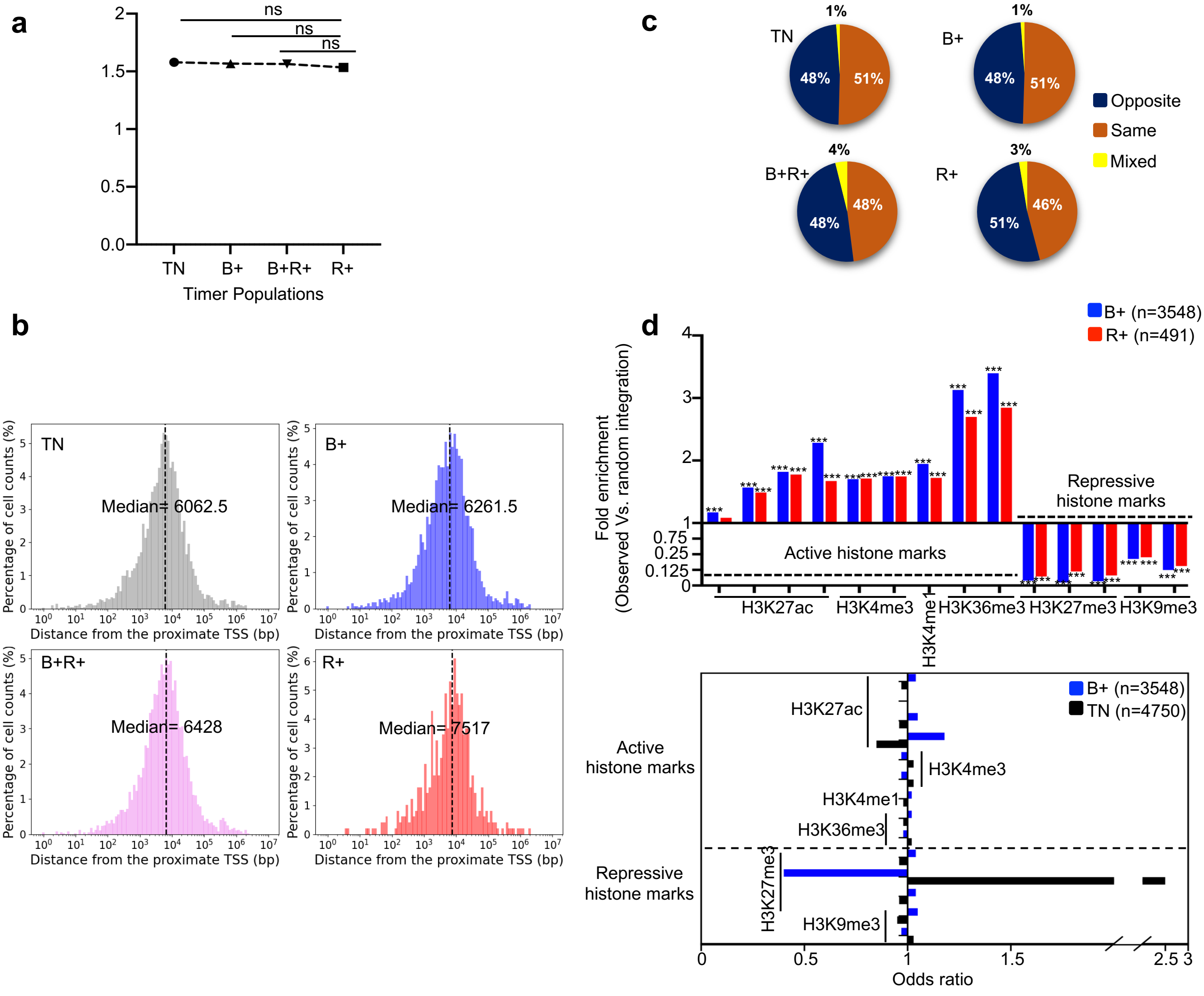

Supplementary Fig. 6. Integration site tendencies of various Timer populations

a Genes of integration from each Timer sorted population (TN, B+, B+R+, and R+) were checked for their basal (pre-integration) TPM values from Jurkat un-infected RNA-seq datasets. Mean Log_{10} (TPM+1) values were calculated and plotted for each Timer population. Statistical significance was calculated by t-test between (TN and R+), (B+ and R+), and (B+R+ and R+). **b** Histograms show the distribution of ISs within each Timer population in relation to the chromosomal distance to the most proximal TSS; the median value is shown for each population. Statistical significance was calculated by Steel-Dwass's multiple comparison test. **c** Pie chart showing percentages of the same or opposite orientation of provirus integration relative to host gene in each Timer population. **d** Upper panel: Integration frequencies near the active histone marks including H3K27ac, H3K4me3, H3Kme1, and H3K36me3, and the repressive histone marks with H3K27me3 and H3K9me3. Integration sites within 2 kb of each histone mark were compared to random expected values. Fold enrichment is represented as the ratio of observed sites/random expected sites. Lower panel: The odds ratio of viral integration sites within ±2 kb of the same histone marks in the upper panel comparing TN and B+. Statistical significance was assessed by Fischer's exact test. *<.05, **<.01, ***<.001.

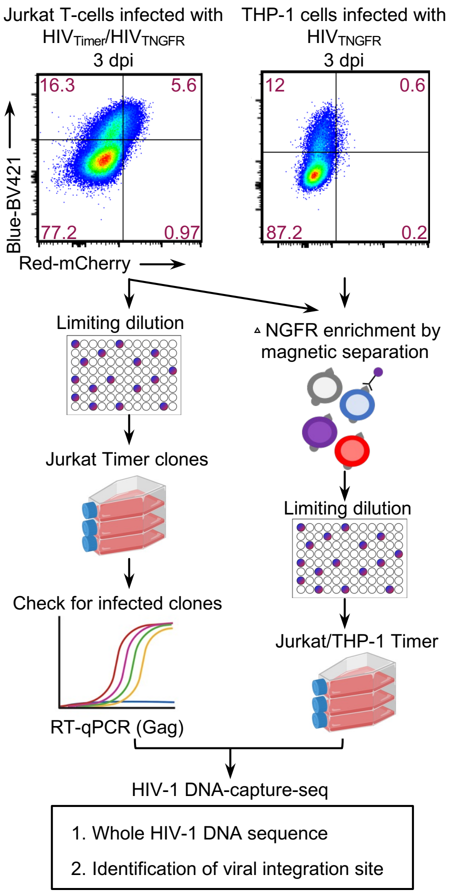

Supplementary Fig. 7. Experimental workflow of Timer clone generation with infected Jurkat T cells or THP-1 cells. Jurkat T cells (left side) were infected with HIV$_{Timer}$ and a limiting dilution was performed at 3 dpi. qPCR check for infected clones was performed. Right side: THP-1 cells or Jurkat T cells were infected with HIV$_{TNGFR}$. Beads sorting for NGFR+ cells was performed at 3 dpi. After the clones' propagation; DNA capture sequencing was performed.

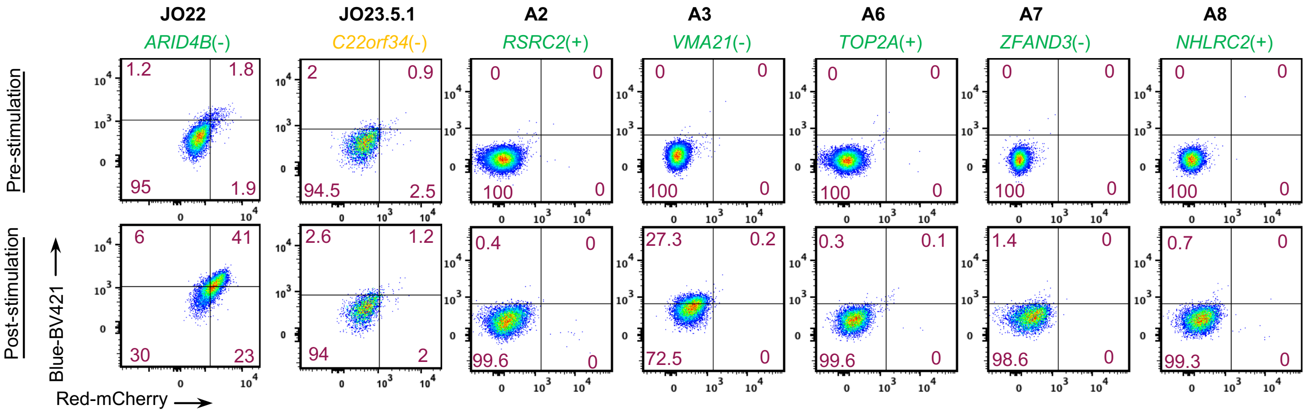

Supplementary Fig. 8. Flow cytometry plots showing Timer-FP expression denoting provirus expression in selected single integration Timer clones. Jurkat Timer clones' basal expression (upper panel); provirus expression after T-cell stimulation for 24 h (lower panel). The integration site of the HIV-1 provirus of each clone is shown under the clone ID. (+) or (-) denotes the same or convergent orientation of provirus integration, respectively.

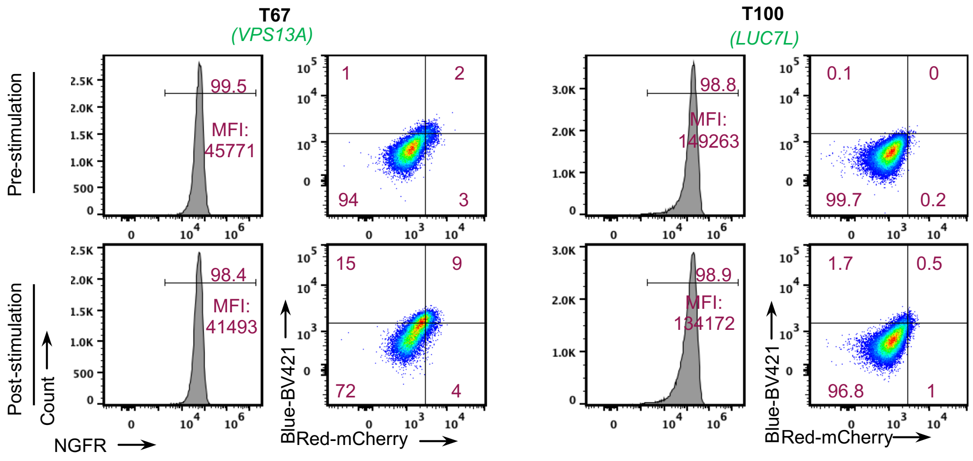

Supplementary Fig. 9. Changes in NGFR expression following Timer clones' activation. Examples from THP-1 Timer clones (shown in Figure 3 c) generated with HIV_{TNGFR} construct infection are shown for basal expression and after stimulation with TNF-α for 24 h.

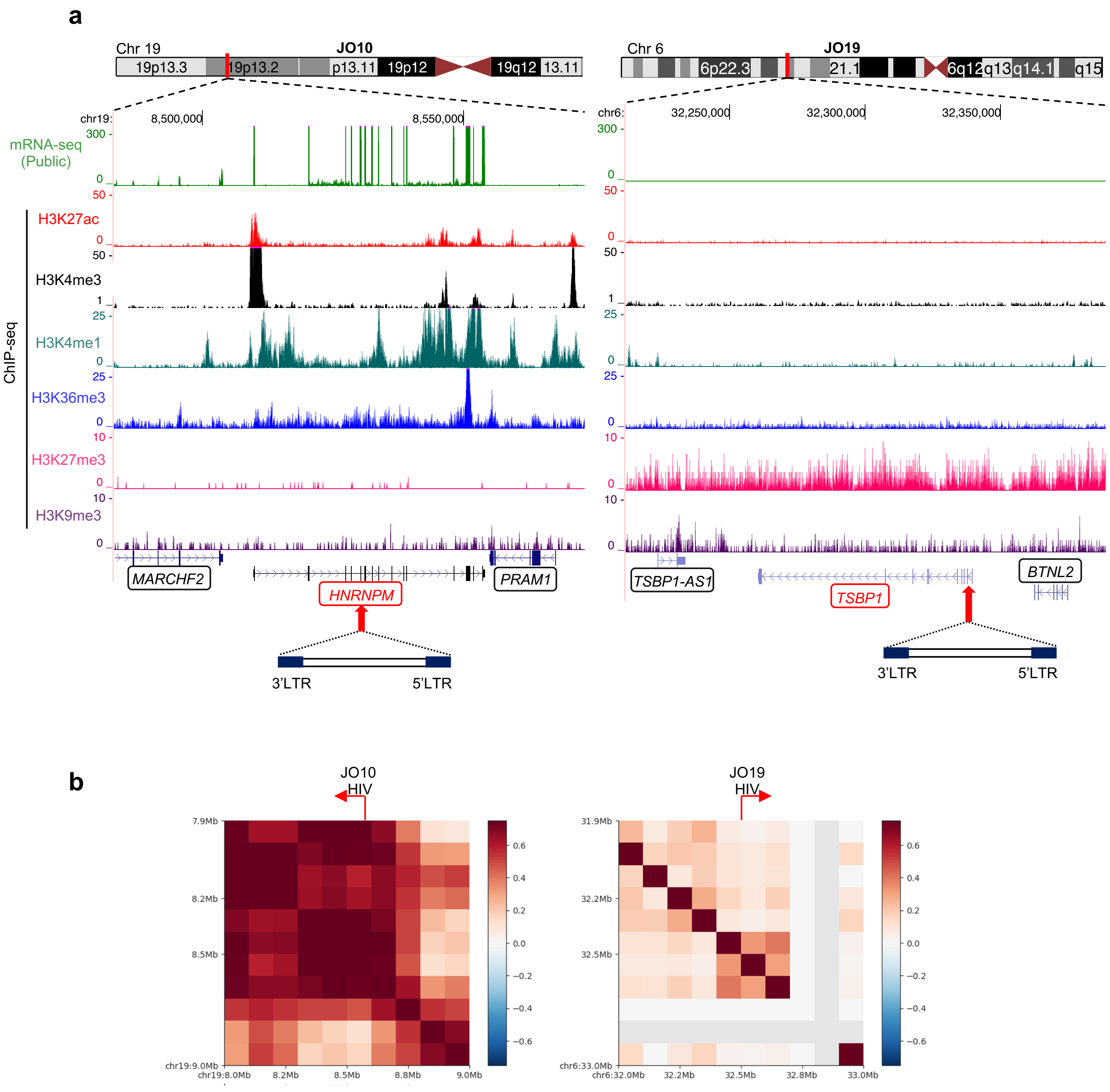

Supplementary Fig. 10. Integration environment of Timer Clones

a RNA-seq data and ChIP-seq data sets of Jurkat T-cell line were plotted by UCSC Genome Browser (https://genome-asia.ucsc.edu) and shown in respect of the integration gene of Timer clones JO10 (left panel) and JO19 (right Panel). Schematic for the position and directionality of provirus integration in each clone is demonstrated at the bottom. **b** Hi-C correlation matrices for Jurkat Timer clones illustrating the correlation (range from blue to red) between the intrachromosomal interaction profiles of every pair of 1-Mb loci along chromosome 19 (Jurkat Timer clone 10; left panel), and chromosome 6 (Jurkat Timer clone 19; right panel).

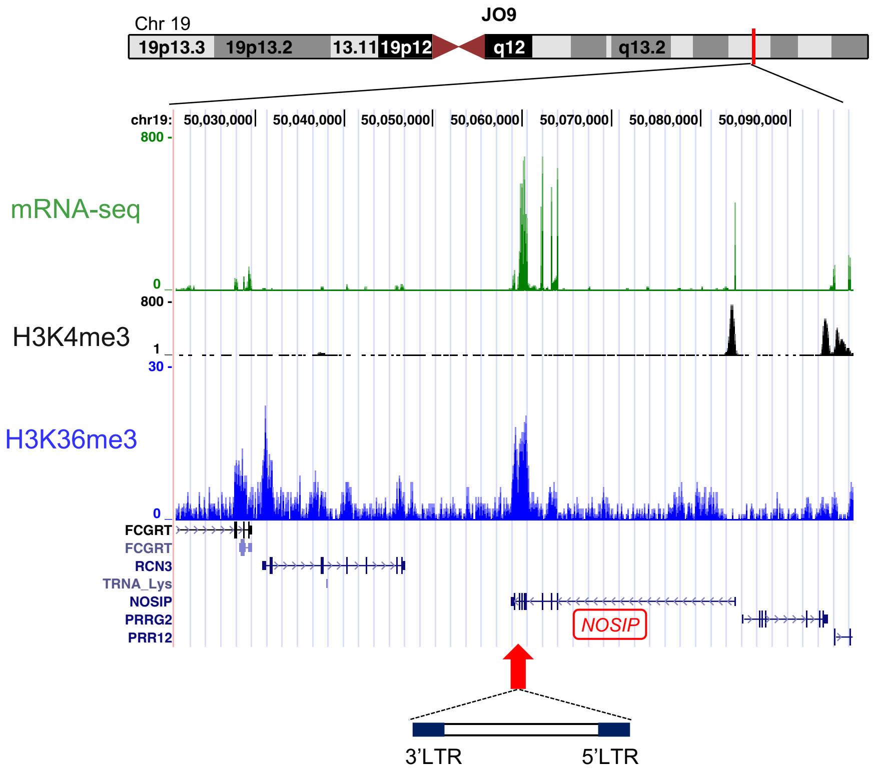

Supplementary Fig. 11. Integration environment of Timer Clone JO9. RNA-seq and ChIP-seq data sets of parent Jurkat T-cell line were plotted by UCSC Genome Browser (https://genome-asia.ucsc.edu) and shown in respect of the integration gene of Timer clone JO9. Schematic for the position and directionality of provirus integration in this clone is demonstrated at the bottom.

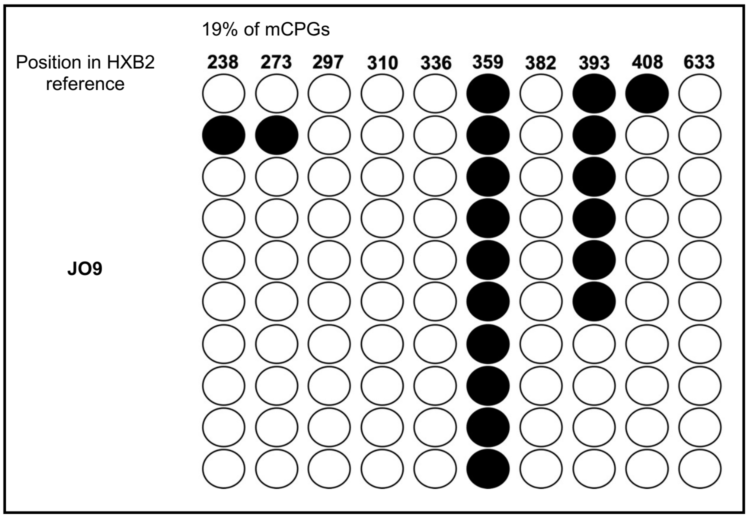

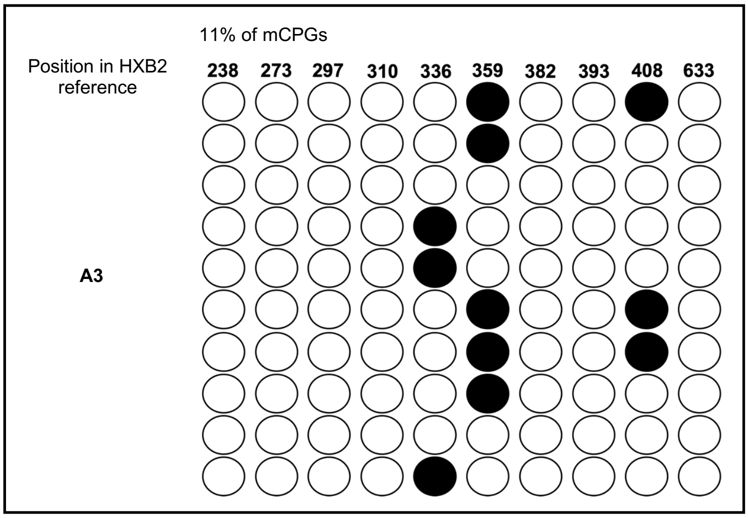

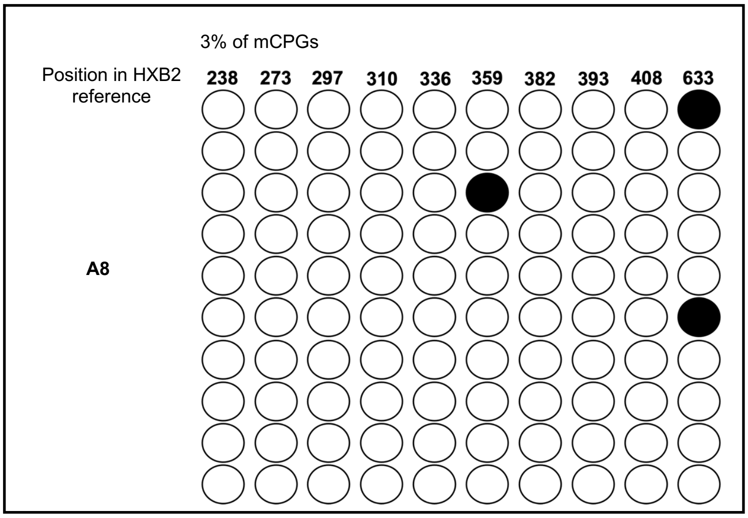

Supplementary Fig. 12. CpG methylation profile of the 5' LTR in Jurkat Timer clones JO9, A3 and A8. Every row is a single genome sequence. Black circles denote methylated CpG residue (mCPGs), open circles denote un-methylated CpG residues. Numbers above each graph denote CpG islands' location within the 5' LTR region. Position 359 is NFAT/ NF- κB - binding site and positions 382,393, and 408 are Sp1-binding sites.

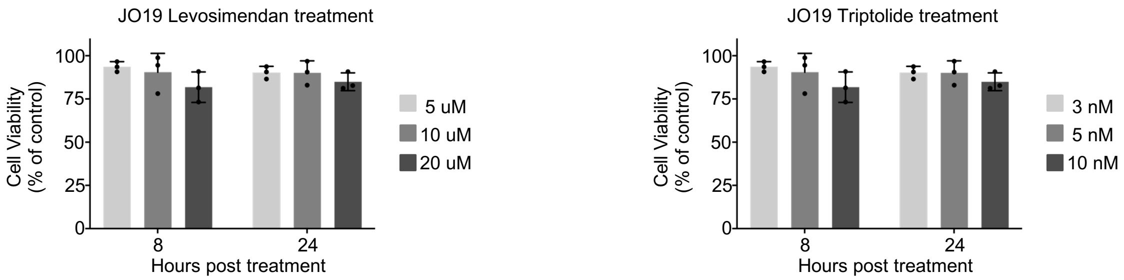

Supplementary Fig. 13. Jurkat T cells or JO19 were treated with Levosimendan, Triptolide, or DMSO control as per shown concentrations for 8 or 24 h. At 8 or 24 h, cells were treated with cell counting kit-8 reagent to check for cell viability. Cell viability % is calculated relative to values from JO19 cells treated with DMSO as control.

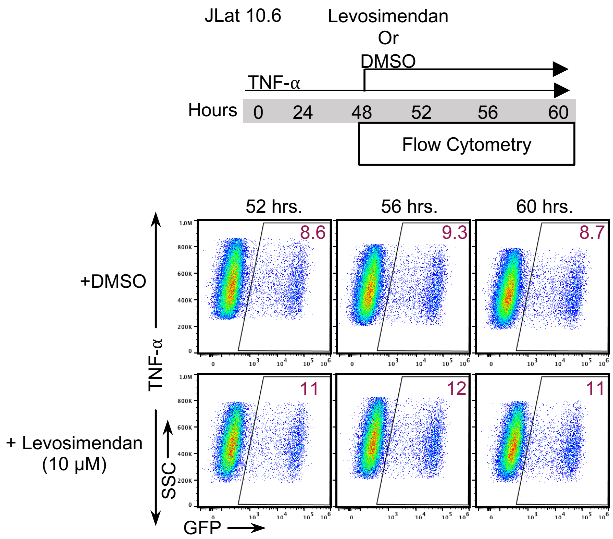

Supplementary Fig. 14. JLat 10.6 cells were stimulated with TNF-α (10 ng/uL) for 48 hrs., an LPA drug (10 μM) or DMSO was further added for 12 h, and the changes in GFP expression were monitored. Flow plots demonstrating GFP transitions under Levosimendan (10 μM) treatment are shown.

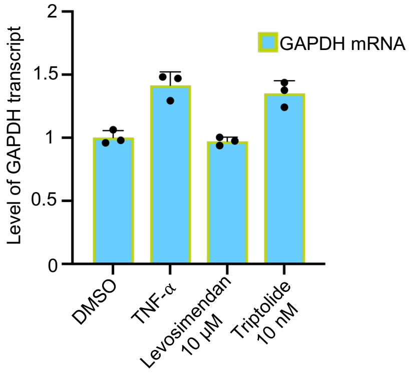

Supplementary Fig. 15. Changes in cellular transcription in comparison to HIV transcription by LPA treatment
Total RNA isolated from Jurkat Clone JO19 cells treated with DMSO or LPA (Levosimendan at 10 μM, and Triptolide at 10 nM) at 12 h post-treatment (shown in Figure 4 D) was subjected to SYBER green RT-qPCR analysis. GAPDH mRNAs were quantified relative to cellular 18s rRNA and the fold change in GAPDH expression was calculated relative to its level in Clone JO19 treated with DMSO. Value from JO19 stimulated with TNF-α for 48h (before LPA treatment initiation) is also shown. (n = 2 biologically independent experiments, mean ± SD).

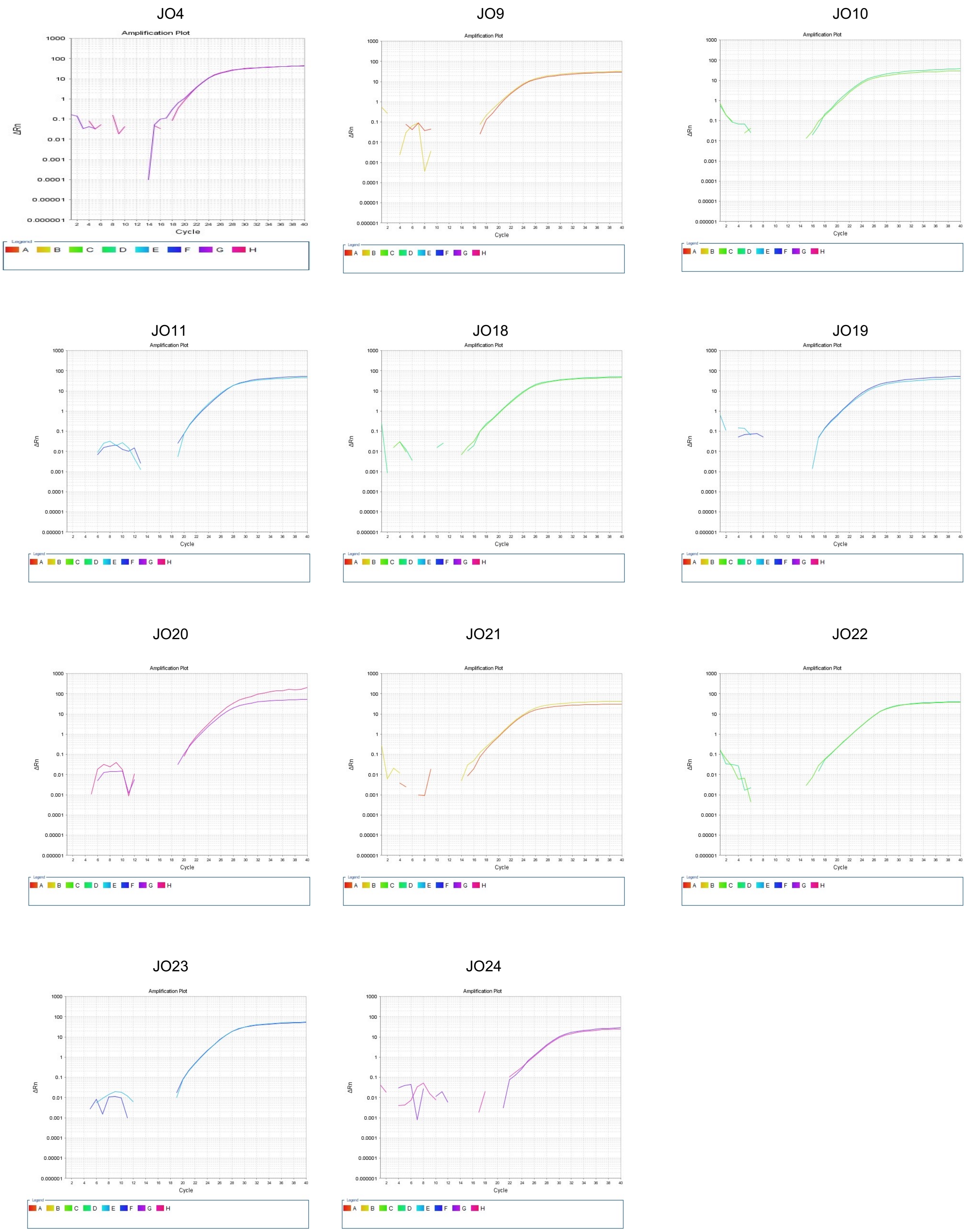

Supplementary Fig. 16. Gag amplification curve by qRT PCR to detect infected Jurkat clones generated by HIV_{Timer} construct..
