# Supplementary material for: HIV-Tocky system to visualize proviral expression dynamics
Source: Commun Biol. 2024 Mar 20;7:344. doi: 10.1038/s42003-024-06025-8 (PMC10954732; doi:10.1038/s42003-024-06025-8)
Supplement: Supplementary file 5 — Reporting Summary [file 42003_2024_6025_MOESM5_ESM.pdf]

nature portfolio

Corresponding author(s): Yorifumi Satou

Last updated by author(s): Feb 21, 2024

Reporting Summary

Nature Portfolio wishes to improve the reproducibility of the work that we publish. This form provides structure for consistency and transparency in reporting. For further information on Nature Portfolio policies, see our Editorial Policies and the Editorial Policy Checklist.

Statistics

For all statistical analyses, confirm that the following items are present in the figure legend, table legend, main text, or Methods section.

n/a	Confirmed	
☐	☒	The exact sample size (*n*) for each experimental group/condition, given as a discrete number and unit of measurement
☐	☒	A statement on whether measurements were taken from distinct samples or whether the same sample was measured repeatedly
☐	☒	The statistical test(s) used AND whether they are one- or two-sided<br>*Only common tests should be described solely by name; describe more complex techniques in the Methods section.*
☒	☐	A description of all covariates tested
☒	☐	A description of any assumptions or corrections, such as tests of normality and adjustment for multiple comparisons
☐	☒	A full description of the statistical parameters including central tendency (e.g. means) or other basic estimates (e.g. regression coefficient) AND variation (e.g. standard deviation) or associated estimates of uncertainty (e.g. confidence intervals)
☐	☒	For null hypothesis testing, the test statistic (e.g. *F*, *t*, *r*) with confidence intervals, effect sizes, degrees of freedom and *P* value noted<br>*Give P values as exact values whenever suitable.*
☒	☐	For Bayesian analysis, information on the choice of priors and Markov chain Monte Carlo settings
☒	☐	For hierarchical and complex designs, identification of the appropriate level for tests and full reporting of outcomes
☒	☐	Estimates of effect sizes (e.g. Cohen's *d*, Pearson's *r*), indicating how they were calculated

Our web collection on statistics for biologists contains articles on many of the points above.

Software and code

Policy information about availability of computer code

Data collection

HIV integration site analysis (LM-PCR):
LM-PCR libraries were prepared by amplifying the junction between 3' LTR of HIV-1 and host genomic DNA in bulk-infected Jurkat cells with the HIVTimer molecular construct. Libraries were sequenced on Illumina MiSeq to obtain paired-end reads. Around 1 million reads were obtained using the following read length: read1_75 bp; read2_75 bp.

DNA-capture-seq:
In samples with HIV-1 probe-capture, libraries were sequenced on Illumina MiSeq to obtain paired-end reads. Around 1 million reads were obtained using the following read length: read1_75 bp; read2_75 bp.

mRNA-seq:
For Timer clones, libraries were sequenced on Illumina NextSeq 550 to obtain single-end reads. Around 40 million reads were obtained using the following read length: read1_75 bp.

ChIP-seq:
Public datasets were used for that section. Data set accession numbers are specified under the methods section: ChIP seq Datasets, epigenetic data analysis, and Hi-C.

Hi-C:
Public datasets were used for that section. Data set accession numbers are specified under the methods section: ChIP seq Datasets, epigenetic data analysis, and Hi-C.

Bisulfite Cytosine Methylation Analysis
Amplified 5'LTR products from the bisulfite-treated Jurkat Timer clones JO9, A3, and A8 were sent for Sanger sequencing to obtain information on the CpG methylation status of the 5'LTR of each provirus from each clone. Bisulfite sequencing fasta files' accession numbers are specified under the data availability section.

Data analysis

FastQC (version 0.10.0) – Checking quality of fastq file (https://www.bioinformatics.babraham.ac.uk/projects/fastqc/)
cutadapt (version 1.18) – Removing the adapter sequences (https://cutadapt.readthedocs.io/en/stable/index.html)
PRINSEQ-lite (version 0.20.4) – Cleaning the sequence data (http://prinseq.sourceforge.net/)
BWA (version 0.7.12) – Alignment for LM-PCR, DNA-capture-seq, ChIP-seq (https://sourceforge.net/projects/bio-bwa)
STAR (version 2.7.3) – Alignment for mRNA-seq (https://github.com/alexdobin/STAR)
SAMTools (version 1.11) – Operating sam and bam files (http://www.htslib.org/download/)
picard (version 2.0.1) – PCR replicate removal (https://broadinstitute.github.io/picard/)
kallisto (version 0.46.1) – a program for quantifying abundances of transcripts from RNA-Seq data (https://github.com/pachterlab/kallisto)
IGV (version 2.15.4) – Used to visualize mRNA-seq, and ChIP-seq data (https://software.broadinstitute.org/software/igv/download)
R (version 4.1.3) – R environment for all custom code
GraphPad Prism 10 – The validation of Statistical significance
FlowJo (version 10.8.1) – FACS analysis
hiAnnotator – R package for integration sites (ISs) distribution analysis (http://github.com/malnirav/hiAnnotator)
MACS (version 1.4.2) – peakcalling of ChIP-seq (https://github.com/macs3-project/MACS)
UCSC Genome Browser- Interactive tool to visualize genomic data (https://genome-asia.ucsc.edu/)
sratoolkit.2.9.4-1- for downloading and converting FASTQ files from public datasets
FAN-C- pipeline for analyzing Hi-C data (https://github.com/vaquerizaslab/fanc)
Cooler: Hi-C data visualization (https://github.com/mirnylab/cooler)
Geneious Prime 2023.2.1

For manuscripts utilizing custom algorithms or software that are central to the research but not yet described in published literature, software must be made available to editors and reviewers. We strongly encourage code deposition in a community repository (e.g. GitHub). See the Nature Portfolio guidelines for submitting code & software for further information.

Data

Policy information about availability of data

All manuscripts must include a data availability statement. This statement should provide the following information, where applicable:

- Accession codes, unique identifiers, or web links for publicly available datasets
- A description of any restrictions on data availability
- For clinical datasets or third party data, please ensure that the statement adheres to our policy

-HIV integration site data, DNA-cap-seq data and Bisulfite-seq data:
Data accession codes are specified under the data availability section.
-ChIP-seq and Hi-C data:
Data used in the manuscript were obtained from public datasets with accession numbers available in the methods section within the manuscript body.

Research involving human participants, their data, or biological material

Policy information about studies with human participants or human data. See also policy information about sex, gender (identity/presentation), and sexual orientation and race, ethnicity and racism.

Reporting on sex and gender	N/A
Reporting on race, ethnicity, or other socially relevant groupings	N/A
Population characteristics	N/A
Recruitment	N/A
Ethics oversight	N/A

Note that full information on the approval of the study protocol must also be provided in the manuscript.

Field-specific reporting

Please select the one below that is the best fit for your research. If you are not sure, read the appropriate sections before making your selection.

☒ Life sciences ☐ Behavioural & social sciences ☐ Ecological, evolutionary & environmental sciences

For a reference copy of the document with all sections, see nature.com/documents/nr-reporting-summary-flat.pdf

Life sciences study design

All studies must disclose on these points even when the disclosure is negative.

Sample size	Sample sizes stated for each experiment within the study were chosen to provide sufficient confidence to validate the Timer fluorescence transitions' ability to capture HIV-1 provirus dynamics, to correlate dynamic status with provirus integration tendency using cell line model. We obtained integration site events as much from each Timer population to validate the results.
Data exclusions	No data were excluded from the manuscript.
Replication	All results shown in the manuscript were validated by triplicates.
Randomization	Not relevant
Blinding	Not required for this study

Reporting for specific materials, systems and methods

We require information from authors about some types of materials, experimental systems and methods used in many studies. Here, indicate whether each material, system or method listed is relevant to your study. If you are not sure if a list item applies to your research, read the appropriate section before selecting a response.

Materials & experimental systems

n/a	Involved in the study	
☐	☒	Antibodies
☐	☒	Eukaryotic cell lines
☒	☐	Palaeontology and archaeology
☒	☐	Animals and other organisms
☒	☐	Clinical data
☒	☐	Dual use research of concern
☒	☐	Plants

Methods

n/a	Involved in the study	
☐	☒	ChIP-seq
☐	☒	Flow cytometry
☒	☐	MRI-based neuroimaging

Antibodies

Antibodies used	All are stated in the manuscript under materials and methods section.
Validation	All antibodies used in the manuscript are well-established by manufactures and utilized in other publications.

Eukaryotic cell lines

Policy information about cell lines and Sex and Gender in Research

Cell line source(s)	Jurkat T cells were obtained from ATCC (E6.1).<br>HEK293T cells were obtained from ATCC (CRL-3216).<br>THP-1 cells obtained from Dr. Hiroaki Takeuchi (Gohda J. et al., Sci Rep. 2018)<br>JLat10.6 were obtained from the National Institutes of Health AIDs reagent Program.
Authentication	None of the cell lines used were authenticated.
Mycoplasma contamination	All cell lines tested negative for mycoplasma contamination.
Commonly misidentified lines (See ICLAC register)	None of the cell lines used in this study are listed in this database.

Plants

Seed stocks	N/A
Novel plant genotypes	N/A
Authentication	N/A

ChIP-seq

Data deposition

☒ Confirm that both raw and final processed data have been deposited in a public database such as GEO.

☒ Confirm that you have deposited or provided access to graph files (e.g. BED files) for the called peaks.

Data access links<br>*May remain private before publication.*	All ChIP-seq datasets used in the manuscript are from public datasets. Accession numbers are stated in the manuscript under methods section.
Files in database submission	H3K27ac (SRR12884765, SRR1603650, SRR1509753, SRR2043614), H3K4me3 (SRR577482, SRR577483), H3K4me1 (SRR7782877), H3K36me3 (SRR11783979, SRR11783980), H3K27me3 (SRR12884766, SRR647929, SRR11903008) and H3K9me3 (SRR13191912, SRR12884767).
Genome browser session<br>(e.g. UCSC)	N/A

Methodology

Replicates	N/A
Sequencing depth	N/A
Antibodies	N/A
Peak calling parameters	MACS with default parameters.<br>Command line: macs callpeak -t [IS_experiment.bam] -c [input_original.bam] -f BAM -g hs -n [output_name]
Data quality	The quality of raw data fastq files were determined with FastQC.<br>Peaks were called with a 5 % FDR threshold.
Software	cutadapt (version 1.18) – Removing the adapter sequences (https://cutadapt.readthedocs.io/en/stable/index.html)<br>PRINSEQ-lite (version 0.20.4) – Cleaning the sequence data (http://prinseq.sourceforge.net/)<br>BWA (version 0.7.12) – Alignment for ChIP-seq (https://sourceforge.net/projects/bio-bwa)<br>SAMTools (version 1.11) – Operating sam and bam files (http://www.htslib.org/download/)<br>picard (version 2.0.1) – PCR replicate removal (https://broadinstitute.github.io/picard/)<br>IGV (version 2.15.4) – Used to visualize ChIP-seq data (https://software.broadinstitute.org/software/igv/download)<br>MACS (version 1.4.2) – peakcalling of ChIP-seq (https://github.com/macs3-project/MACS)

Flow Cytometry

Plots

Confirm that:

☒ The axis labels state the marker and fluorochrome used (e.g. CD4-FITC).

☒ The axis scales are clearly visible. Include numbers along axes only for bottom left plot of group (a 'group' is an analysis of identical markers).

☒ All plots are contour plots with outliers or pseudocolor plots.

☒ A numerical value for number of cells or percentage (with statistics) is provided.

Methodology

Sample preparation	Primary CD4+ T-cells isolation and infection<br>Primary CD4+ T-cells were extracted from healthy donors' PBMCs using density centrifugation on Ficoll-Paque Plus gradient (cytiva, Cat# 17144002). Resting CD4+ T-cells were enriched by negative depletion in two successive steps, first labeling with Biotin anti-human CD8 a (BioLegend, Cat # 301004) and Biotin-TCR γ/δ Antibody (BioLegend, Cat#331206) negative and secondly adding Pan T cell Isolation cocktail (Miltenyi Biotec, Cat#17144002) according to manufacturer instructions. Isolated cells were then cultured in RPMI with 10% fetal bovine serum, penicillin (100 U/ml), and streptomycin (100 ug/ml) with additional recombinant human IL-2 (PEPROTECH, Cat#200-02) at a final concentration of 100 U/ml. Next, cells were activated with Dynabeads Human T-Activator aCD3/CD28 activating beads (gibco by Life Technologies, Cat# 11131D) at a concentration of 1 bead/cell in the presence of IL-2 for two days. We infected primary CD4+ T-cells using the RetroNectin-Bound Virus infection method (TaKaRa, Cat#T100A/B) according to the manufacturer's instructions. HIVTimer construct was used for infection at a concentration of 500 ng of p24 per 1×106 cells. Cells were collected at 1 dpi until 6 dpi and analyzed by flow cytometry.<br>Timer flow cytometric analysis and cell sorting<br>For integration site analysis, Jurkat T cells were infected with the HIVTimer virus construct as described above. Infected cells (5×105) were aliquoted at 12 h, and 1–7 dpi and cryopreserved. Time-course flow cytometric analysis was performed by

thawing and analyzing all time points in the same setting. For Timer-only fluorescence detection (For infected primary CD4+ T-cells and Jurkat T-cells), cells were revived in pre-warmed complete RPMI 1640 medium, washed once with PBS, and incubated in near-IR fluorescent live/dead fixable dye (Thermo Fischer, Cat# L34972) to stain dead cells at a 1:1000 dilution in PBS for 30 min at 4 degree C. Cells were then washed once and then fixed with 1% PFA in PBS for 15 min at 4 degree C in the dark before data acquisition. To determine the surface NGFR expression along with Timer, revived cells were first stained for dead cells as described above, and after a single wash in PBS, they were stained with APC-conjugated anti-human CD271 (NGFR) antibody (BioLegend, Cat# 345108) at 1:30 final concentration for 30 min at 4 degree C. Cells were washed twice with PBS and fixed in 1% PFA in PBS for 15 min in dark at 4 C before data acquisition. Timer and NGFR fluorescence were measured and/or sorted using SH800 sorter (Sony). Timer Blue fluorescence was detected in the FL1 channel (450/50 nm) excited by a 405 nm laser, while the red fluorescence was detected in the FL3 channel (617/30) excited by a 561 nm laser. Data were analyzed using FlowJo 10.7.1 software (Tree Star, Inc.). For viral mRNA quantification, Jurkat T cells were infected with the HIVTNGFR virus construct as described above. The infected cells were collected from day one to 7 post-infection and cryopreserved. Aliquots were then resuspended in warm complete RPMI 1640 medium, washed once with PBS, and stained with near-IR fluorescent live/dead fixable dye (Thermo Fischer, Cat# L34972), followed by staining with APC-conjugated anti-human CD271 (NGFR) antibody (BioLegend, Cat # 345108) at 1:30 final concentration as described above. Cells were finally washed twice with PBS and resuspended in 1% FCS in PBS buffer. The Timer populations were sorted using BD FACSAria III machine at the bio-containment level 3 facility at Kumamoto University.

Instrument

Sony sorter (SH800 sorter) and BD FACs ARIA IIIu.

Software

Data acquisition:
-Sony sorter : SH800S software.
-BD FACs ARIA IIIu: BD FACSDiva
Data analysis:
FlowJo 10.7.1 software (Tree Star, Inc.).

Cell population abundance

To obtain enough cells for downstream analysis, TN and R+ cells were sorted from 1 and 4 dpi, respectively. B+ and B+R+ cells were sorted on days 2 and day 3 post-infection samples. Post sort purity was not below 75%. Purity check plots are shown in the SI file. (Suppl. Fig. 3a).

Gating strategy

In case of infected cells with HIV-Timer construct: Infected cells were gated by forward scatter (FSC) vs. side scatter (SSC). Viable cells were gated by APC-Cy7 viability dye peak. Timer quadrants gating was performed using non-infected control.
In case of infected cells with HIV-TNGFR: Infected cells were gated by forward scatter (FSC) vs. side scatter (SSC). Viable cells were gated by APC-Cy7 viability dye peak. Then, Infected cells were gated by APC-NGFR peak from viable cells. Timer quadrants gating was performed using non-infected control.

☒ Tick this box to confirm that a figure exemplifying the gating strategy is provided in the Supplementary Information.
